# The effect of the difference in C_2–7_ angle on the occurrence of dysphagia after anterior cervical discectomy and fusion with the zero-P implant system

**DOI:** 10.1186/s12891-020-03691-7

**Published:** 2020-10-06

**Authors:** Cheng-Yi Huang, Yang Meng, Bei-Yu Wang, Jie Yu, Chen Ding, Yi Yang, Ting-Kui Wu, Hao Liu

**Affiliations:** 1 Department of Orthopedic Surgery, West China Hospital, Sichuan University, No. 37 Guo Xue Xiang, Chengdu, 610041 Sichuan China; 2 Department of Spinal Surgery, Beijing Jishuitan Hospital, The 4th Clinical Medical College of Peking University, No. 31, Xinjiekou East Street, Xicheng District, Beijing, 100035 China

**Keywords:** Dysphagia, Zero-P, Cervical vertebrae, Related factors

## Abstract

**Objectives:**

To investigate the effect of the difference in C_2–7_ angle on dysphagia after anterior cervical discectomy and fusion (ACDF) with the Zero-P Implant System.

**Methods:**

A retrospective analysis of 181 patients who underwent ACDF with the Zero-P Implant System and had at least one year of follow-up from January 2011 to November 2018 was performed. All patients were divided into a non-dysphagia group and a dysphagia group to explore the effect of the difference between postoperative and preoperative C_2–7_ angle (dC_2–7_A) on postoperative dysphagia. At the same time, other possible related factors including the difference between postoperative and preoperative O-C2 angle (dO-C2A), sex, age, body mass index (BMI), intraoperative time, estimated blood loss, diabetes mellitus, hypertension, smoking, alcohol consumption, prevertebral soft-tissue swelling (PSTS), the highest segment involved in the surgery and the levels of surgery segments were analyzed.

**Results:**

In total, the non-dysphagia group comprised 139 patients and the dysphagia group comprised 42 patients. The single-factor analysis showed that smoking, PSTS and dC_2–7_A were significantly different between the two groups (*P* < 0.05). Spearman’s correlation coefficient showed no significant correlation between the degree of dysphagia and dC_2–7_A (*P* > 0.05). The results of the multiple-factor analysis with an ordinal logistic regression model showed that smoking, PSTS and dC_2–7_A were significantly associated with the incidence of dysphagia (P < 0.05).

**Conclusions:**

The postoperative C_2–7_ angle has an important effect on the occurrence of dysphagia in patients undergoing Zero-P implant system interbody fusion surgery.

## Introduction

Anterior cervical discectomy and fusion (ACDF) has become a widely accepted and practiced surgical intervention for the treatment of cervical spondylosis and disc herniation [1, 2]. Traditional ACDF has been applied with an anterior titanium alloy plate in cervical spine surgery to maintain or increase the alignment and stability of the cervical sagitta, increase the fusion rate, and decrease the potential for graft extrusion and subsidence, particularly in surgery involving multiple segments [1–4]. Unfortunately, the utilization of the anterior plate significantly increases the risk of dysphagia after surgery, and this procedure is often complicated by the collapse of the grafted bone, pseudarthrosis, kyphotic deformity, and graft donor site morbidity [5–7]. To reduce the incidence of complications such as dysphagia, a new stand-alone zero-profile device (Zero-P, Synthes GmbH, Switzerland) is commonly used for ACDF in our hospital. Although the Zero-P Implant System has been confirmed to reduce the incidence of complications to a considerable degree, many patients suffer from dysphagia after surgery [8–10]. Some previous studies have conducted risk factor analyses of postoperative dysphagia after traditional ACDF [1, 5], but few studies have focused on the related factors of postoperative dysphagia in ACDF with the Zero-P Implant System.

We noticed that after the ACDF with Zero-P, the changes in lordosis in some patients were obvious, especially the C2–7 angle; these changes could cause changes in the anatomical relationship between the cervical spine and the anterior esophagus, which may cause dysphagia. Several retrospective studies have reported that the change in O-C_2_A plays an important role in the development of dysphagia after occipitocervical fusion [11, 12]. A retrospective study based on 392 patients showed that the change in C_2–7_ angle plays an important role in the development of dysphagia in both ACDF with anterior plate and posterior cervical laminoplasty [13]. However, few reports have described the effect of the difference between postoperative and preoperative C_2–7_ angle (dC_2–7_A) on postoperative dysphagia after ACDF with Zero-P.

Thus, considering the paucity of clinical data in this field, a retrospective analysis of patients who underwent ACDF with the Zero-P Implant System was performed in our center to investigate the effect of the dC_2–7_A and other possible related factors on dysphagia. Practical references are provided for future surgeries to further reduce the incidence of dysphagia after ACDF with Zero-P.

## Materials and methods

This retrospective study was approved by the Medical Ethics Committee of West China Hospital, Sichuan University, China. All of the patients were recruited after providing informed consent for analysis of their clinical data.

### Inclusion and exclusion criteria for patients

#### Inclusion criteria

① Patients with radiculopathy or myelopathy from single-level or multilevel cervical disc disease with correlating magnetic resonance imaging findings and no response to conservative treatment for more than 6 weeks;② patients aged over 18 years who underwent ACDF with the Zero-P Implant System from C_3_ to C_7_;③ patients with detailed postoperative anteroposterior and lateral X-rays and clinical data; and④ patients who had accepted at least one year of follow-up.

#### Exclusion criteria

① Patients with dysphagia before the surgery; ②patients with psychological diseases such as mania and severe depression or a history of central nervous system disorders such as traumatic brain injury and brain stroke;③ patients who had a history of esophageal disease, revision surgery and neck surgery; and ④ patients with pathologic fractures of the vertebrae, spinal deformity, ossification of the posterior longitudinal ligament (OPLL), rheumatoid arthritis, ankylosing spondylitis, developmental stenosis and local or systemic infections.

This retrospective review was conducted with 181 patients who underwent ACDF with the Zero-P Implant System between January 2011 and November 2018 in West China Hospital, Sichuan University, Sichuan, China. All patients were treated, nursed and followed up in the Department of Orthopedics, West China Hospital.

### Surgical technique

All surgical procedures were performed by senior spinal surgeons in our department with a standard, right Smith-Robinson approach after the induction of general anesthesia [14, 15]. A transverse incision from the midline to the anterior border of the sternocleidomastoid was used to reach the perpendicularly spreading fibers of the platysma, and then the platysma was split longitudinally. Complete discectomy was performed at the index levels by removing the disc tissue, posterior longitudinal ligament and osteophytes to achieve thorough decompression. After the endplates were well prepared, a properly sized Zero-P implant filled with a composite synthetic bone graft (beta-tricalcium phosphate, β-TCP, ChronOS; DePuySynthes, Paoli, CA, USA) was implanted into the index levels. The locking head screws were screwed into place after final imaging of the device was performed. Layer-by-layer suturing and incision closure were performed after drainage insertion. All patients received methylprednisolone at a dosage of 100 mg per day during the 3 days after the surgery; meanwhile, rhBMP-2 was not used.

### Dysphagia evaluation

The Bazaz grading system was chosen to evaluate dysphagia after surgery because it has been commonly used in previous studies [16]. The Bazaz grading system is a qualitative, unvalidated grading scale used to obtain patients’ perception of their difficulty swallowing liquids and solids based on individualized information. The scores of the Bazaz grading system were ranked as follows: 0-none, 1-mild, 2-moderate and 3-severe, representing no episodes of swallowing problems, rare episodes of dysphagia, occasional swallowing difficulties with specific foods and frequent swallowing difficulties with most foods, respectively. In this study, the grade of dysphagia was evaluated by investigators via telephone interviews or outpatient follow-up visits based on the information provided by the patients, and the patients were promptly evaluated for dysphagia 1 week after surgery and at each follow-up point. Patients were divided into two groups according to the presence or absence of dysphagia (Table 1).

**Table 1:** The Bazaz grading system

Severity	Liquids	Solids
0-None	None	None
1-Mild	None	Rare
2-Moderate	None or rare	Occasionally
3-Severe	None or rare	Frequent

### Radiographic assessment

**Fig. 1:**
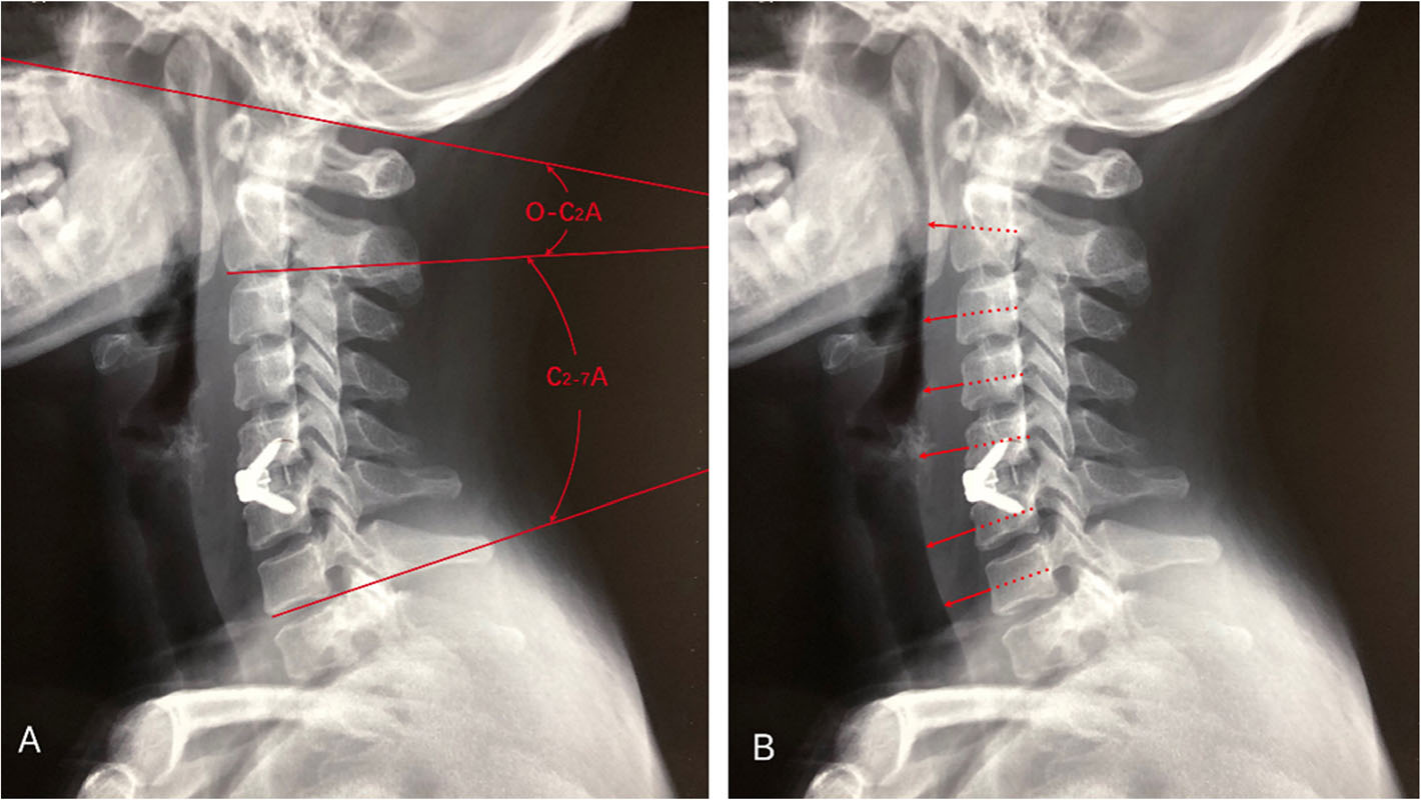
The measurement of O-C_2_A, C_2–7_A(**a**) and PSTT(**b**) on postoperative lateral X-rays. The angle between the inferior margin of the C_2_ vertebrae and the inferior margin of the C_7_ vertebrae was measured as the C_2–7_ angle while the angle between the McGregor’s line and the inferior margin of the C_2_ vertebrae was measured as the O-C2 angle perioperatively on plain lateral cervical radiographs (**a**). The PSTT was measured at the extended line of the AP diameter from C_2_ to C_7,_ and the AP diameter was measured between the center of the posterior and anterior cortex of each vertebral body. O-C_2_A: O-C_2_ angle, C_2–7_A: C_2–7_ angle, AP: Anteroposterior, PSTT: Prevertebral soft tissue thickness

PACS version 4.0 (GE Healthcare, Milwaukee, WI) was used for measuring the O-C2 angle (O-C_2_A), C_2–7_ angle (C_2–7_A) and prevertebral soft-tissue thickness (PSTT). The angle between the inferior margin of the C_2_ vertebrae and the inferior margin of the C_7_ vertebrae was measured as the C_2–7_ angle [17, 18] while the angle between the McGregor’s line and the inferior margin of the C_2_ vertebrae was measured as the O-C2 angle perioperatively on plain lateral cervical radiographs [19]. The prevertebral soft tissue thickness was measured at the extended line of the anteroposterior (AP) diameter which ends at the posterior aspect of the trachea from C_2_ to C_7_, and the AP diameter was measured between the centers of the posterior and anterior cortex of each vertebral body (Fig. 1) [20]. The average of the PSTT from C_2_ to C_7_ was taken for analysis. The difference between the postoperative and preoperative O-C_2_ angle (dO-C_2_A), the difference between the postoperative and preoperative C_2–7_ angle (dC_2–7_A) and prevertebral soft-tissue swelling (PSTS) were calculated from the following formulas: dO-C_2_A = postoperative O-C_2_A − preoperative O-C_2_A, dC_2–7_A = postoperative C_2–7_A − preoperative O-C_2_A, and PSTS = postoperative PSTT−preoperative PSTT. The measurement time of the X-rays was 1 week after surgery, and two independent radiologists performed the assessment of the X-rays

### Statistical analysis

All statistical analyses were performed with the statistical program SPSS version 22.0 (SPSS Inc., Chicago, IL, USA). The investigators used descriptive statistics such as the means, standard deviations and ratio index to represent the quantitative and categorical variables. For the single-factor analysis, a chi-squared test and Student’s t-test were conducted for enumeration and measurement data, respectively. Spearman’s correlation coefficient was calculated between the degree of dysphagia and dC_2–7_A. To eliminate the influence of confounding factors, ordinal logistic regression was performed for multifactor regression of factors with a *P* value less than 0.2 in the single-factor analysis. The results were regarded as significant when the *P*-values were less than 0.05 in this study.

## Results

In total, 181 consecutive patients were enrolled in this study according to the inclusion and exclusion criteria listed above, including 104 males and 77 females with ages ranging from 25 to 77 years and an average age of 52.15 ± 9.32 years. The dysphagia group consisted of 42 consecutive patients while the non-dysphagia group consisted of 139 consecutive patients. The difference in preoperative C_2–7_A, O-C_2_A and PSTT between the two groups was not significant (*P* > 0.05), whereas the postoperative C_2–7_A, O-C_2_A and PSTT were significantly different between the two groups (*P* < 0.05) (Table 2). The mean follow-up period was 18 months (range: 12 to 24 months).

**Table 2:** The pre- and postoperative radiographic parameters

Groups	Non-dysphagia group (*N* = 139)	Dysphagia group(*N* = 42)	*P*
Preoperative O-C_2_ angle (°)	16.14 ± 6.77	15.36 ± 6.56	0.510
Postoperative O-C_2_ angle (°)	15.78 ± 3.60	14.46 ± 3.48	**0.037**
Preoperative C_2–7_ angle (°)	15.02 ± 11.61	15.40 ± 7.92	0.843
Postoperative C_2–7_ angle (°)	12.14 ± 5.23	19.77 ± 6.17	**< 0.001**
Preoperative PSTT (mm)	9.39 ± 1.53	11.65 ± 1.88	0.386
Postoperative PSTT (mm)	14.91 ± 4.06	18.06 ± 3.07	**< 0.001**

### Single-factor analysis between the postoperative C_2–7_ angle and dysphagia

**Fig. 2:**
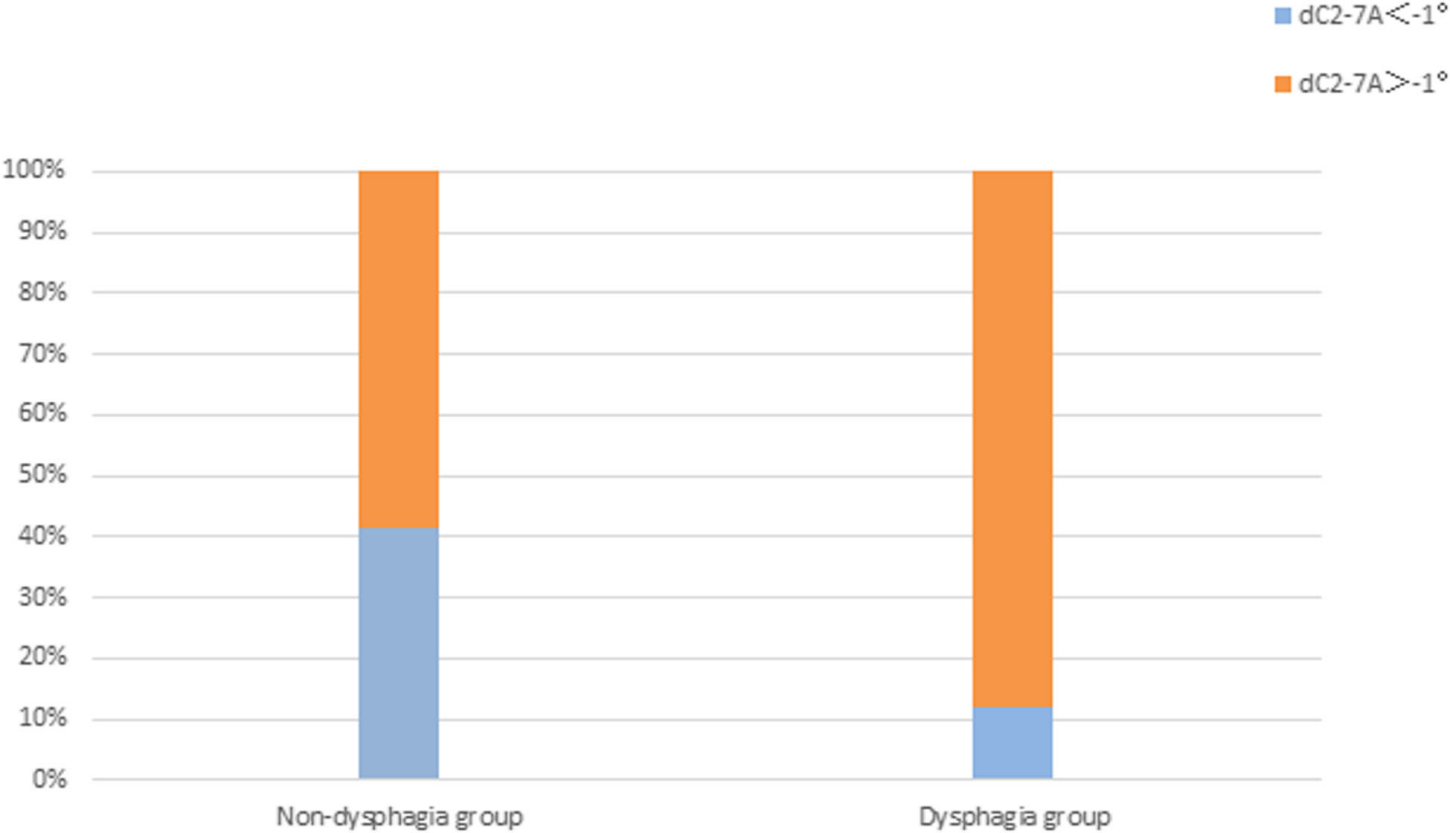
Comparison of the dC_2–7_A between non-dysphagia and dysphagia groups. dC_2–7_A, difference between postoperative and preoperative C_2–7_ angle

According to the assessment and statistical analysis of the radiographs, we evaluated the role of dC_2–7_A in the development of dysphagia and found a significant difference in dC_2–7_ angle for dysphagia and non-dysphagia patients in the two groups. (*P* < 0.01, Table 3). Moreover, 63 of the 181 patients had a postoperative dC_2–7_A < -1°, dysphagia occurred in 5 of those patients, and the incidence rate was 7.94% (5/63). Meanwhile, 118 of the 181 patients had a postoperative dC2–7 A ≥ -1°, dysphagia occurred in 37 of those patients, and the incidence rate was 31.36% (37/118). The difference was significant (*P* < 0.05) (Fig. 2).

**Table 3:** Comparison of factors of dysphagia after surgeries

Groups	Non-dysphagia group(*N* = 139)	Dysphagia group (*N* = 42)	*P*
Sex (male/female)^b^	78/61	26/16	0.506
Age (y)^a^	52.71 ± 9.98	50.31 ± 6.41	0.145
BMI^a^	23.75 ± 2.92	24.67 ± 3.29	0.084
Diabetes mellitus (yes/none)^b^	5/134	2/40	0.732
Hypertension (yes/none)^b^	2/127	5/37	0.524
Smoking (yes/none)^b^	31/108	20/22	**0.001**
Alcohol consumption (yes/none)^b^	45/94	17/25	0.332
Intraoperative time (min)^a^	147.60 ± 17.23	150.43 ± 18.60	0.361
Estimated blood loss (ml)^a^	80.72 ± 78.73	58.10 ± 22.87	0.068
Prevertebral soft-tissue swelling (mm)^a^	5.52 ± 0.67	6.41 ± 0.87	**< 0.001**
dO-C_2_A (°)^a^	−0.37 ± 7.68	−0.91 ± 7.22	0.687
dC_2–7_A (°)^a^	−2.88 ± 9.69	4.37 ± 6.82	**< 0.001**
The highest segment involved in the surgery^a^	6.15 ± 0.79	6.29 ± 0.77	0.332
Number of surgery segments^a^	1.91 ± 0.76	1.81 ± 0.55	0.444

^a^Student’s t-test, ^b^Chi-squared test

In the dysphagia group, 26 patients suffered from mild dysphagia, 9 patients suffered from moderate dysphagia, and 7 patients suffered from severe dysphagia according to the Bazaz grading system. Spearman’s correlation coefficient of the dysphagia group between the degree of dysphagia and the incidence of postoperative dysphagia showed no significant correlation (r = 0.051, *P* = 0.747).

### Single-factor analysis between other related factors and dysphagia

Table 3 shows that the distribution of age, sex and BMI was similar in the dysphagia and non-dysphagia groups (*P* > 0.05). The prevalence of diabetes mellitus and hypertension was not significantly different between the two groups (*P* > 0.05), and similar results were obtained for alcohol consumption (P > 0.05). However, smoking was significantly different between the two groups (*P* < 0.05). The postoperative O-C_2_ angle was similar (P > 0.05), whereas the PSTS in the two groups was significantly different (P < 0.05). The intraoperative time and estimated mean blood loss were not significantly different in the dysphagia and non-dysphagia groups (*P* > 0.05). With regard to the highest segment involve in the surgery, the mean was found to be not significantly different between the dysphagia and non-dysphagia groups (P > 0.05). In addition, the single-level, two-level, and three-level surgeries were denoted by “1”, “2” and “3”, respectively, for the analysis of the levels of surgery, and the levels of both groups ranged from 1 to 3. Between the average level in the dysphagia and non-dysphagia groups, no significant differences were observed (*P* > 0.05).

### Multivariate logistic regression analysis

**Fig. 3:**
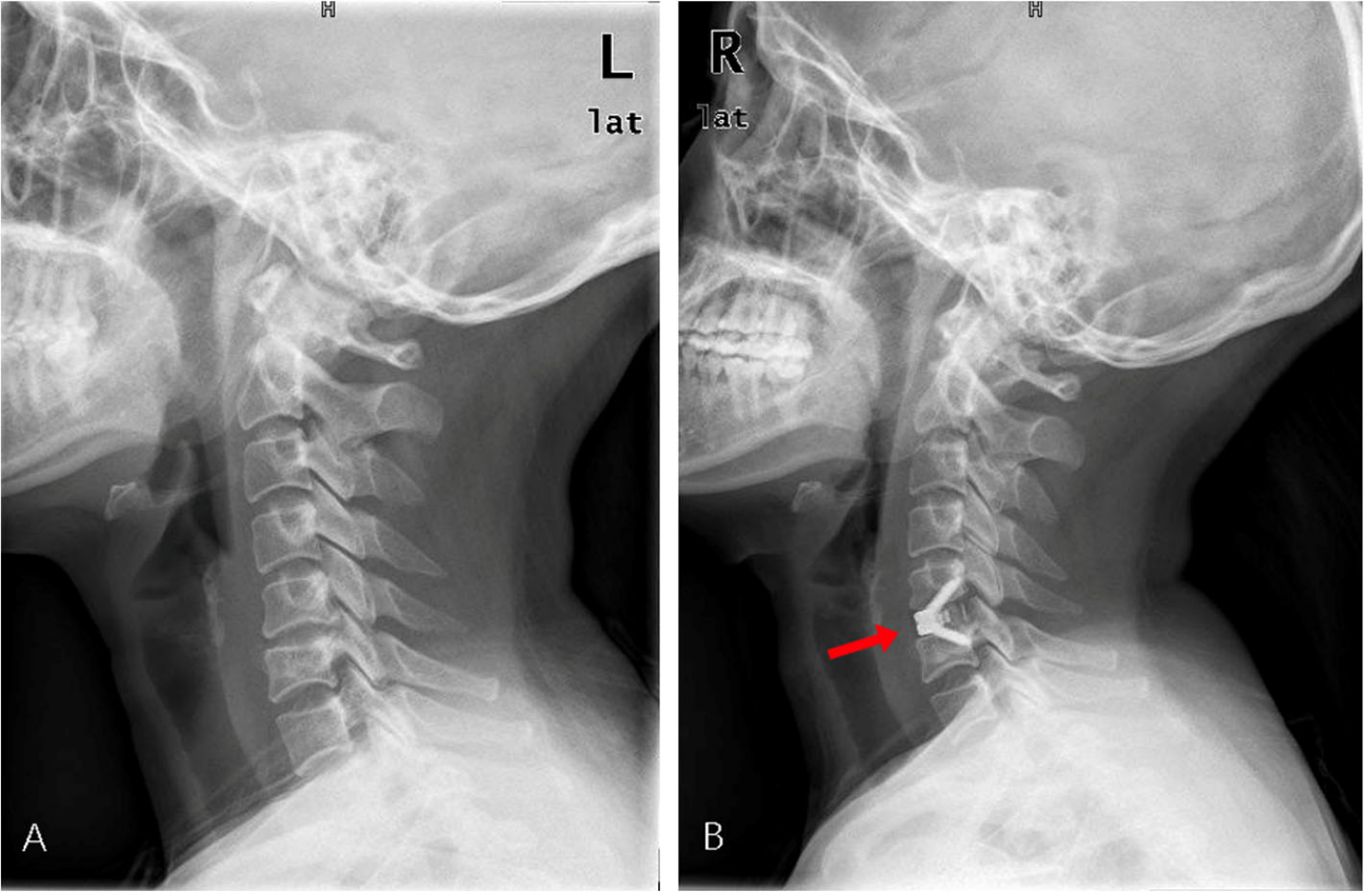
The preoperative (**a**) and postoperative (**b**) lateral X-rays of a 40-year-old female patient who was diagnosed with cervical spondylotic myelopathy and underwent C5/6 ACDF with Zero-P. The dO-C_2_A was 8.62°. The Bazaz grading system showed mild postoperative dysphagia. The X-rays showed obvious dC_2–7_A (red arrow)

**Fig. 4:**
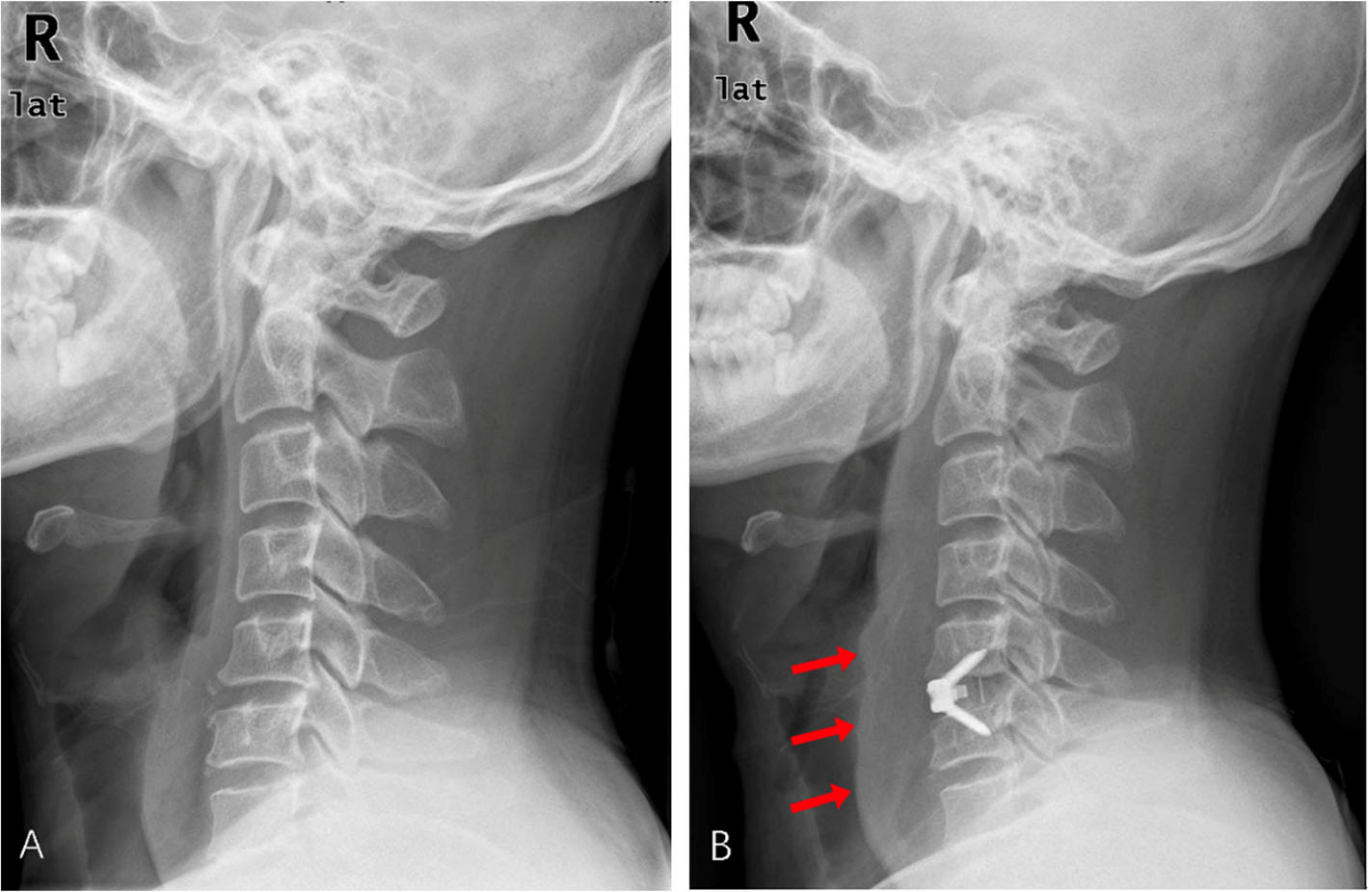
The preoperative (**a**) and postoperative (**b**) lateral X-rays of a 37-year-old male patient who was diagnosed with cervical spondylotic myelopathy and underwent C5/6 ACDF with Zero-P. The PSTS was 6.3 mm. The Bazaz grading system showed severe postoperative dysphagia. The X-rays showed obvious PSTS (red arrows). PSTS: Prevertebral soft-tissue swelling

Ordinal logistic regression was performed for multifactor regression of the factors that had a *P* value less than 0.2 in the single factor analysis, including age, BMI, smoking, estimated blood loss, PSTS and dC_2–7_A. The results showed that smoking, PSTS (Fig. 3) and dC_2–7_A (Fig. 4) were significantly associated with a higher incidence of dysphagia (*P* < 0.05), whereas age, BMI and estimated blood loss were not associated with a higher incidence of dysphagia (*P* > 0.05). The results of the multiple factors analysis are shown in Table 4.

**Table 4:** The results of the logistic regression analysis between related factors and dysphagia^a^

Related factors	B	P	OR	95%CI	
				Lower bound	Upper bound
Age	−0.008	0.776	0.992	0.939	1.048
BMI	0.143	0.077	1.154	0.984	1.353
Smoking	1.769	**0.001**	5.863	2.138	16.082
Estimated blood loss	−0.009	0.126	0.991	0.980	1.002
Prevertebral soft-tissue swelling	1.788	**< 0.001**	5.975	2.891	12.350
dC_2–7_A	0.077	**0.018**	1.080	1.013	1.151

^a^Only the factors with a *P* value less than 0.2 in the single factor analysis were included

## Discussion

Dysphagia, or swallowing dysfunction, can possibly reduce patient satisfaction with surgery, lead to various degrees of discomfort, and increase the risk of various complications such as bronchospasm, aspiration pneumonia, dehydration, asphyxia and malnutrition [21]. Dysphagia is a postoperative result triggered by multiple factors, and it is reported to be the most common early complication after ACDF [21–23]. Rihn et al. [24] concluded that dysphagia appeared among 70% of patients who underwent anterior cervical surgery. With a mean of 7.2 years of follow-up, Yue et al. [25] found varying degrees of dysphagia occurring after ACDF with an anterior cervical plate, with an incidence of 35.1% at the final follow-up.

The mechanism underlying the occurrence of postoperative dysphagia is still controversial. Dysphagia is possibly associated with damage to the swallowing center in the central nervous system or cortical areas, dysfunction of efferent nerves or muscular drive or decreased pharyngolaryngeal sensitivity. Disorders of esophageal motility caused by intra-operative traction or mechanical stimulation during surgery have often been mentioned in previous studies [26]. Swelling of the soft tissue around the esophagus and anterior cervical plate after ACDF are also commonly recognized as causes of postoperative dysphagia [27, 28]. Attempts have been made in recent years to overcome the limitations of the traditional anterior plate. Lee et al. [29] proved that a low-profile plate design can minimize soft tissue irritation, thus decreasing the incidence of dysphagia after traditional ACDF. New devices for ACDF have been developed in recent years, such as the Zero-P Implant System. In theory, the Zero-P Implant System reduces the incidence of dysphagia after surgery due to the lack of posterior irritation and constriction by anterior plates.

Several retrospective studies have reported that the change in lordosis plays an important role in the development of dysphagia in both anterior and posterior cervical spine surgery [11–13]. Tian et al. [13] found that dC_2–7_A for dysphagia patients was significantly higher than for non-dysphagia patients after ACDF. In general, dC_2–7_A could cause changes in the anatomical relationship between the cervical spine and the anterior esophagus, which may cause dysphagia. However, the traditional anterior cervical plate can better reconstruct the overall curvature of the cervical spine and the curvature of the surgical segment than the Zero-P [30, 31]. Studies focusing specifically on the effect of the dC_2–7_A on the development of dysphagia after ACDF with the Zero-P are rare. Therefore, we designed this retrospective study.

In this study, the comparison of the dC_2–7_A between the dysphagia group and the non-dysphagia group showed that the difference was significant. Meanwhile, the incidence of dysphagia when the dC_2–7_A was <− 1° was obviously less than when the dC_2–7_A was ≥ − 1°. A greater dC_2–7_A was shown to be significantly associated with a higher incidence of dysphagia. Therefore, adjusting the C_2–7_ angle properly in ACDF with the Zero-P Implant System may reduce the incidence of dysphagia. Some surgeons exert more powerful traction on the prevertebral tissue and intervertebral space to restore the physiological curvature of the cervical spine, decrease postoperative cervical degeneration and create a larger space for the implant, and some insert the screws more smoothly during the operation; ignoring this may lead to an angle that is too large, resulting in the protrusion of the pharyngolaryngeal wall and, ultimately, postoperative dysphagia [32]. Notably, Spearman’s correlation coefficient between the degree of dysphagia and dC_2–7_A showed no significant correlation in the dysphagia group. We therefore can assume that the dysphagia is a type of subjective feeling, which might be different for similar anatomical changes after surgery.

We noticed that the risk factors for dysphagia after ACDF with Zero-P varied greatly in different studies. Miyata et al. [11] and Meng et al. [12]. reported that the difference between postoperative and preoperative O-C_2_ is a key factor in the development of postoperative dysphagia after occipitocervical fusion. Kalb et al. reported that possible risk factors included multilevel surgeries, the involvement of C_4–5_ and C_5–6_, and age but not operating time in their study [33]. Jang et al. concluded that age and sex were not related to postoperative dysphagia [34]. Elderly age, female sex and multilevel surgery were found to be possible risk factors for postoperative dysphagia in the study by Zeng et al. [35]. Some factors, such as the change in O-C2 angle, sex, age, levels of surgery segments and the highest segment of surgery, were not associated with a higher incidence of dysphagia in this study. Several reasons for this are possible. First, the Zero-P Implant System can reduce the incidence of dysphagia after surgery compared with traditional ACDF because of the absence of an anterior cervical plate, possibly resulting in the lack of a significant difference between the two groups with the highest segments involved in the surgery and the levels of surgery. Second, the common application of the anterior cervical soft-tissue spreader reduces the intraoperative traction on the esophagus; injury to the recurrent laryngeal nerve (RLN) and superior laryngeal nerve (SLN) is also avoided due to the improvements in anterior cervical surgery in recent years. In addition, cultural factors in different regions may affect the risk factors for dysphagia. Notably, PSTS had an important effect on the occurrence of dysphagia in this study, consistent with previous findings [36, 37]. This might be because the intra-operative traction of esophagus and trachea, split of prevertebral fascia, stimulation of implants or even the change in cervical physiological curvature can cause prevertebral soft-tissue swelling leading to dysphagia. Paying attention to minimizing the intra-operative traction and routine use of corticosteroids after surgery is necessary according to the result. Furthermore, smoking was significantly associated with a higher incidence of dysphagia in the ordinal logistic regression model in this study, and this has rarely been mentioned before. Possible reasons are that smoking can cause pharyngolaryngitis and increase the sensitivity of the pharynx and larynx. Studies with larger sample sizes are possibly needed to validate the authenticity of this result.

### Limitation

The limitations of the study are as follows. First, adoption of the Bazaz scale is the primary limitation of this study. As an unvalidated grading scale used to evaluate dysphagia, this scale is based on qualitative information collected by an investigator to assess the patient’s subjective sensation of difficulty when swallowing liquids and solids; this scale has been commonly applied in previous studies [16]. As the scale is based on subjective feelings, possible sensory disruptions causing postoperative dysphagia may be challenging to explain and may not reflect accurate clinical outcomes. The gold standard of dysphagia assessment, which is fiber optic endoscopic evaluation or video fluoroscopy, could be used to ensure the veracity of these findings [38, 39]. Second, the limitations of the retrospective design are obvious; therefore, future randomized controlled studies are also needed to verify our conclusions. Third, not all potential risk factors, such as the changes in cervical curvature, were considered in the statistical analysis. In addition, the mechanisms by which the dC_2–7_A affects the development of dysphagia after ACDF with the Zero-P Implant System are not completely clear. Therefore, multi-center and randomized controlled studies are needed to verify our conclusions in the future.

## Conclusion

The difference between the postoperative and preoperative C_2–7_ angle has an important effect on the occurrence of dysphagia in patients undergoing Zero-P implant system interbody fusion surgery. Measurement and adjustment of the C_2–7_ angle during ACDF with Zero-P may be practical and essential in avoiding inadvertent postoperative dysphagia. Further randomized controlled studies are needed to validate these findings.

## Data Availability

The datasets used and/or analysed during the current study are available from the corresponding author on reasonable request.

## Abbreviations

ACDF: Anterior cervical discectomy and fusion
Zero-P: Zero-profile standalone device
O-C2A: O-C2 angle
C2–7A: C2–7 angle
dC2–7A: Difference between postoperative and preoperative C2–7 angle
dO-C2A: Difference between postoperative and preoperative O-C2 angle
PSTT: Prevertebral soft tissue thickness
PSTS: Prevertebral soft-tissue swelling
BMI: Body mass index
OPLL: Ossification of the posterior longitudinal ligament
rhBMP-2: Recombinant human bone morphogenetic protein-2
AP: Anteroposterior
RLN: Recurrent laryngeal nerve
SLN: Superior laryngeal nerve
